# AAV-based vectors for human diseases modeling in laboratory animals

**DOI:** 10.3389/fmed.2024.1499605

**Published:** 2025-02-12

**Authors:** Timur I. Aliev, Dmitry V. Yudkin

**Affiliations:** ^1^Department of Natural Sciences, Novosibirsk State University, Novosibirsk, Russia; ^2^Federal State Autonomous Educational Institution of Higher Education I.M. Sechenov First Moscow State Medical University of the Ministry of Health of the Russian Federation (Sechenov University), Moscow, Russia

**Keywords:** adeno-associated virus, transgenesis, animal models, human diseases, gene therapy

## Abstract

The development of therapeutic drugs and vaccines requires the availability of appropriate model animals that replicate the pathogenesis of human diseases. Both native and transgenic animals can be utilized as models. The advantage of transgenic animals lies in their ability to simulate specific properties desired by researchers. However, there is often a need for the rapid production of transgenic animal models, especially in situations like a pandemic, as was evident during COVID-19. An important tool for transgenesis is the adeno-associated virus. The genome of adeno-associated virus serves as a convenient expression cassette for delivering various DNA constructs into cells, and this method has proven effective in practice. This review analyzes the features of the adeno-associated virus genome that make it an advantageous vector for transgenesis. Additionally, examples of utilizing adeno-associated viral vectors to create animal models for hereditary, oncological, and viral human diseases are provided.

## 1 Introduction

Recombinant adeno-associated virus (rAAV) has proven to be an effective system for delivering transgenes into living organisms, particularly in humans (1). This effectiveness is largely due to the fact that many natural serotypes used in transgenesis and gene therapy have been isolated from human tissues and non-human primates (NHPs), which are the native hosts of AAV. AAV-based vectors have several advantages over other viral vectors. Firstly, each serotype possesses its own tissue tropism, allowing researchers to select specific cell types for transduction, thereby reducing transgene expression in off-target tissues. For example, AAV1 has the highest tropism for mammalian skeletal muscle, AAV3 targets human and NHP hepatocytes, and AAV9 has a unique ability to cross the blood–brain barrier and the blood-testis barrier, enabling transduction of the mammalian central nervous system (CNS) and mammalian germ cells, respectively (2–4). Secondly, even wild-type AAV (wtAAV) is not pathogenic to humans and does not cause any diseases. Its replication is only possible in the presence of helper viruses (herpesviruses, adenoviruses, papillomaviruses, bocaviruses) (5). In the absence of a helper virus, the AAV genome integrates into the AAVS1 locus, which serves as a safe harbor in humans and NHPs (6). This integration does not disrupt the functions of endogenous genes. Thirdly, rAAV and wtAAV exhibit low immunogenicity compared to common adenovirus and lentivirus vectors (7, 8). Fourthly, AAV vectors provide long-term expression of the transgene (9). Fifth, the ability to pseudotype AAV allows for an expansion of tissue tropism and a reduction in immune response to seroprevalent serotypes (10, 11). This capability enables the application of AAV vectors not only for routine transgenesis but also for gene therapy targeting human genetic disorders. As of April 2024, the U.S. Food and Drug Administration (FDA) has approved seven AAV vector-based therapies for gene therapy, including the well-known Glybera, Luxturna, and Zolgensma (1). The ability of AAV to effectively deliver transgenes into living organisms is also applied in generating transgenic animal models for various biological tasks, including the modeling of different human diseases and conditions. The classical approach to obtaining transgenic animals involves electroporation or pronuclear or intracytoplasmic microinjection of the zygote, followed by embryo transfer to pseudopregnant females. This approach allows for the generation of stable strains with consistent transmission of genetic modifications. However, it requires a significant amount of time and cannot be used in situations with limited time constraints. Unlike classical methods of animal transgenesis, the approach using AAV is less time- and labor-intensive. Moreover, it enables the production of two types of transgenic animals. The first type exhibits the desired trait as a result of transient transgene expression, while the second type carries either a transgene that is integrated into the genome or a knockout. The molecular mechanisms for achieving the target effects vary: overexpression, RNA interference, and gene knockout. In this review, we analyze the features of the AAV genome that make it a convenient tool for animal transgenesis and provide examples of the use of AAV vectors to create animal models simulating human diseases with various etiologies.

## 2 The features of the AAV genome make it a convenient tool for transgenesis

The AAV genome acts as an expression cassette for effective transgenesis. To deliver the transgene, the single-stranded DNA of the wild-type AAV (wtAAV) genome undergoes to various manipulations using genetic engineering methods. The wtAAV genome contains two genes, *rep* and *cap*, flanked by inverted terminal repeats (ITRs). In recombinant AAV (rAAV) vectors, these genes are replaced with cargo, which includes the gene of interest (GOI), promoter, polyA site, etc. (6). The transgene cassette, which must not exceed the size of the AAV genome – 4.7 kb – does not contain any viral genome sequences except for the ITRs. The ITRs are essential for AAV genome replication, encapsidation, and the conversion of the single-stranded genome into a double-stranded genome for stable transgene expression. The Rep-binding element (RBE), located in each ITR, is necessary for binding with the Rep proteins and introducing single-strand breaks at the terminal resolution sites (trs) during replication, which is crucial for separating the two single-stranded AAV genomes (12). A transgenic cassette, flanked by ITRs, can be cloned into any plasmid with a selective marker for production at the necessary scale in a bacterial system. The utilized helper plasmids encode all the necessary helper factors for the assembly of viral particles: serotype-specific capsid proteins (cap gene, encoding VP1, VP2, VP3, MAAP, and AAP), the viral replicative complex (rep gene, encoding Rep78, Rep68, Rep52, and Rep40), as well as the E2A and E4 genes of adenovirus, and the virus-associated RNA of adenovirus (VA RNAI) (5). An undeniable advantage for virus production is the availability of the HEK293 cell line and its variants, which express the E1A and E1B genes of human adenovirus 5 (13). The proteins encoded by these genes are non-toxic to cells. AAV-based vectors, isolated from producer cells and purified, can be administered to laboratory animals via injection into the bloodstream or any other tissue, depending on the purpose. AAV vectors then penetrate cells through interactions with serotype-specific receptors located on the cell surface. For example, for AAV2, AAV3, AAV6, and AAV13, the primary receptor is heparan sulfate proteoglycans (HSPG); for AAV9, it is N-linked glycans with terminal galactosyl residues (referred to as terminal N-linked galactose); and for AAV1, AAV4, AAV5, and AAV6, sialic acids serve as the receptors (14). For some serotypes, secondary receptors (also known as co-receptors) have been identified, including laminins, integrins, platelet-derived growth factor receptor (PDGFR), fibroblast growth factor receptor (FGFR), and hepatocyte growth factor receptor (HGFR/C-MET) (15). After successful internalization, the AAV vector in the form of an endosome is delivered to the nucleus via the microtubule system using the motor protein dynein (16). After import into the cell nucleus, the single-stranded transgene cassette is replicated using host cell enzymes of matrix synthesis. Viral ITRs form structures that serve as primers for replication. The resulting double-stranded AAV genome forms episomes, providing prolonged episomal expression.

However, over time, transgene expression in cells decreases. This decline is due to the inability of the episome to self-replicate, the low frequency of AAV vector integration into the genome, and regular cell proliferation, especially in tissues with a high capacity for regeneration. It has been shown that after the introduction of AAV8 and AAVrh10 vectors into the saphenous vein, the level of transgene expression in the liver of rhesus macaques decreased fourfold compared to the peak value over 98 days (17). Analysis of liver biopsies using the ITR-seq method showed instances of integration at a frequency of 1 viral genome per 100 cells. Such random integrations do not ensure stable expression. Therefore, when stable and long-term expression is required, the integration of the transgene into the genome via genome editing tools (such as CRISPR-Cas9) is necessary. However, the packaging capacity of a single AAV-based system does not allow it to carry both the gene of interest (GOI) and the genomic editing tools for integration simultaneously. These limitations necessitate the creation of a hybrid system, where one AAV vector carries the GOI for integration, while the second carries the necessary tools. In 2013, the first prototype of a hybrid-vector system was introduced, which utilizes transposase-mediated somatic integration for stabilized transgene expression (18). In this system, the first AAV vector contains a Tc1/mariner transposon with the neomycin resistance gene controlled by the SV40/TN5 promoter, and the second vector delivers the hyperactive Sleeping Beauty (SB) transposase SB100X under the control of the CMV promoter. In this case, AAV vectors serve as direct substrates for transposition. Simultaneous transduction of HeLa cells using both AAV vectors led to successful integration of the transgene cassette into the genome. Evaluation of integration events using a plasmid rescue strategy and a linear amplification-mediated PCR (LAM-PCR) protocol showed that integration sites were located in both introns and exons, indicating random integration regardless of chromatin structure. This hybrid-vector system has been widely used as a platform for providing stable transgene expression levels throughout the lifetime for studying the effectiveness of gene therapy for hereditary diseases in animal models (19–21).

However, with this approach, the number and location of insertions are not regulated. Additionally, the expression level of the transgene is influenced by the chromatin state at the integration site. As a result, there is a risk of insertional mutagenesis due to integrations in functionally significant loci, and there can be varying levels of expression in cells depending on the transgene copy number (22). To avoid negative consequences, integration can be targeted to specific loci known as “safe harbors.” Genomic safe harbors are regions in the genome that can support stable transgene expression without disrupting host cell functions, as confirmed in numerous studies (23). For transgene integration, the *Rosa26* locus is widely used; it has orthologs in mice, rats, rabbits, bovines, primates, including humans (24–26). It has been shown that AAV vectors can serve as donor DNA for homologous recombination (HR). This allowed for the integration of a transgene at the site of double-strand DNA breaks mediated by CRISPR-Cas9 activity (27, 28).

In summary, AAV vectors represent a convenient system not only for creating model animals that replicate human disease pathogenesis but also for gene therapy for hereditary diseases. The combination of AAV vectors as a delivery system with genome editing tools and the broad ability to modify various parts of the AAV genome make them a versatile tool. The following section will discuss examples of creating model animals for simulating hereditary, oncological, and infectious diseases.

## 3 Cases of successful generation of animal models using AAV

AAV is capable of infecting not only primates but also other mammals. This is due to the fact that the entry of certain AAV serotypes into cells is mediated by interactions with heparan sulfate proteoglycans (HSPG) and sialic acid, which are widely present on the surface of cells in many mammalian species. Numerous studies have demonstrated the possibility of transducing the tissues of mice, rats, dogs, cats, bovines, and other animals using AAV (29). This allows the use of AAV vectors for transgenesis in laboratory animals. This section presents examples of transgenesis in laboratory animals to create models that reproduce the pathogenesis of human diseases.

### 3.1 Models of hereditary and oncological diseases

#### 3.1.1 Parkinson’s disease

**Figure 1:**
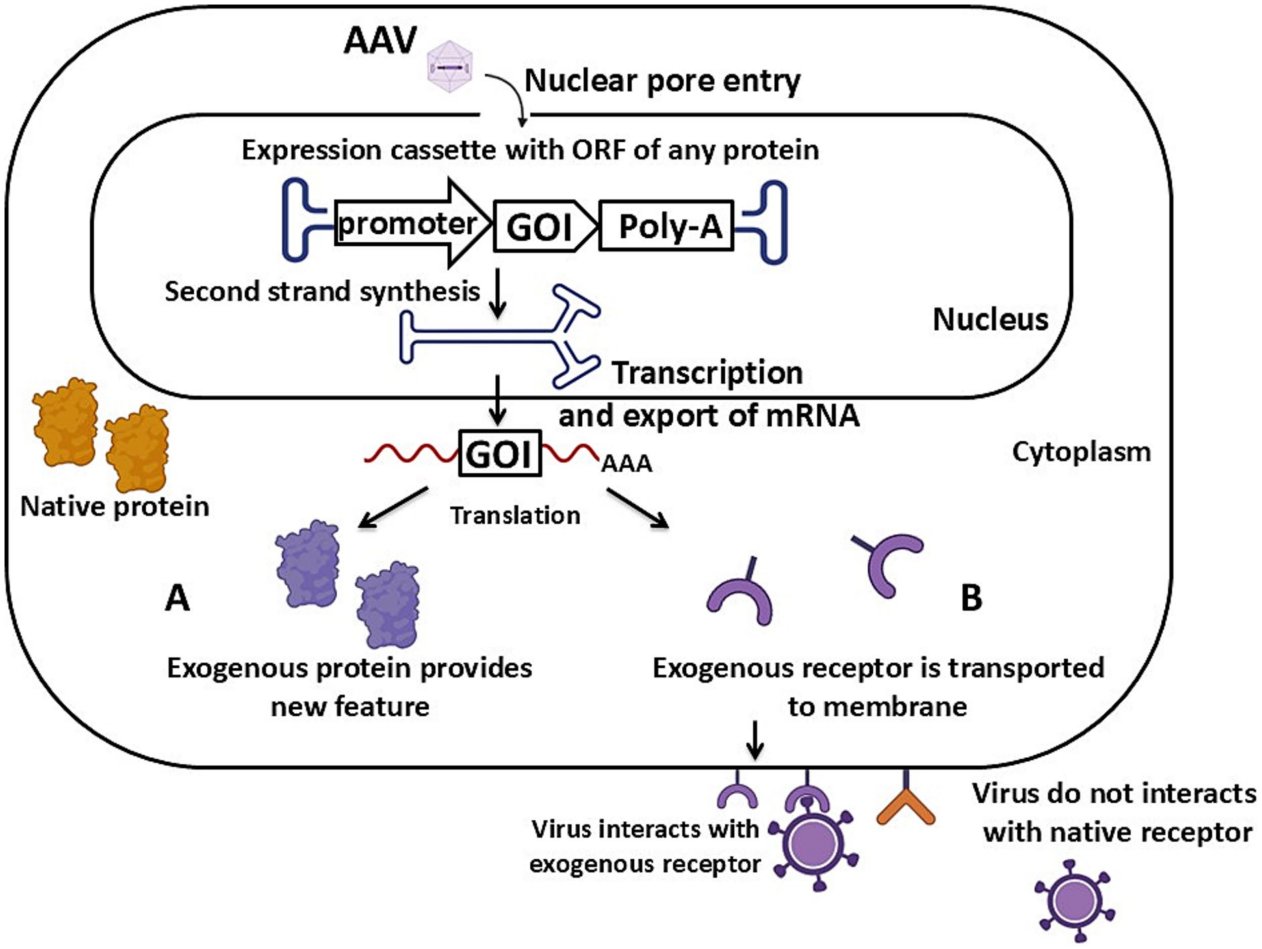
Schematic of AAV vector-mediated transgenesis via the expression mechanism of an exogenous protein. **(A)** The expression cassette carries an ORF of a protein localized in the cytoplasm. **(B)** The expression cassette carries an ORF of a protein localized on the cell’s cytoplasmic membrane. Detailed description in the text. GOI – gene of interest, Poly-A – poly-A signal. Created with BioRender.com.

Parkinson’s disease (PD) is a neurodegenerative disorder characterized by the degeneration of dopaminergic (DA) neurons in the substantia nigra pars compacta and the formation of abnormal Lewy bodies containing aggregates of the protein *α*-synuclein (SNCA, α-syn) (30). It is now clear that mutant *α*-syn takes center stage in PD and plays a key role in the formation of Lewy bodies. Mutations in the SNCA gene encoding α-synuclein, such as A53T, A30P, E46K, H50Q, G51D, and A53E, have been identified. Another gene associated with PD is *LRRK2* (leucine-rich repeat kinase 2) (31). The most prevalent mutation in *LRRK2* is the G2019S substitution, accounting for 5 to 6% of familial PD and 1 to 2% of *de novo* genetic PD cases. LRRK2 and *α*-syn may functionally interact to induce the degeneration of dopaminergic neurons through mechanisms that are not yet fully understood. To confirm that LRRK2 with the G2019S substitution increases the toxicity of mutant A53T *α*-syn when both mutant forms are co-expressed in dopaminergic neurons, rat models mimicking the development of PD have been created (Table 1 and Figure 1A). The G2019S mutation is known to increase LRRK2 kinase activity. To evaluate the role of mutations in functional proteins, they created transgenic cassettes coding different forms of the C-terminal portion of human LRRK2 (aa 1,283–2,527) (LRRK^G2019S^, LRRK^WT^) and full-length human *α*-syn (α-syn^A53T^, α-syn^WT^), both under the PGK1 promoter. AAV6 was used for delivery. To detect neurodegeneration mediated by the mutant form of LRRK2 and α-syn, vectors were injected into the substantia nigra pars compacta (SNpc) of adult Sprague–Dawley rats in various combinations. Behavioral and histological assessments conducted 15 weeks after injection showed that none of the forms of LRRK2 alone induced neuronal degeneration after injection. In contrast, injection of AAV-*α*-syn^A53T^ alone led to motor impairments and degeneration of DA neurons. Co-injection of AAV-*α*-sy*n^A53T^* and AAV-LRRK2^G2019S^ induced DA neuron degeneration that was significantly greater than that induced by AAV-*α*-syn^A53T^ alone or in combination with AAV-LRRK2^WT^. Thus, the obtained model demonstrated that the neurotoxicity of mutant α-syn can be enhanced by the C-terminal portion of human LRRK2^G2019S^.

**Table 1:** Summary of existing transgenic animal models obtained with AAV-based vectors.

Modeled condition	AAV serotype	Animal species and strain	Target organ	Transgene	Modeling mechanism and result	Reference
Models for medical genetics and oncology						
Parkinson’s disease	AAV6	Sprague–Dawley rats	Brain	ORF *hLRRK2* (aa 1,283–2,527 of C-terminal domain) and ORF *hSNCA,* each under control of PGK1 promoter (Figure 1A).	Co-expression of mutant and endogenous forms of LRRK and α-syn resulted in the manifestation of pathogenesis characteristic of PD.	(89)
Spinal muscular atrophy type 1	scAAV9	Pig	Spinal cord	shRNA1 targeting porcine *Smn* under control of the H1 promoter (Figure 2).	Silencing the *Smn* gene using RNA interference led to a reduction in gene expression by 73 ± 6% in motor neurons and 26 ± 10% in the dorsal horn, resulting in a complete loss of motor function.	(34)
Asthma	AAV5	C57BL/6 mice	Upper respiratory tract	shRNA targeting murine *Ctnnal1* (Figure 2).	Silencing the *Ctnnal1* gene using RNA interference led to a 60% reduction in gene expression and, consequently, an exacerbation of asthma symptoms after allergen exposure: mucus hypersecretion, airway inflammation, and increased goblet cell hyperplasia.	(37)
Desmin-related myofibrillar myopathy	AAV1	C57BL/6 mice	Skeletal muscles	ORF of full-length human desmin with R406W or E413K substitution under control of the CMV promoter (Figure 1A).	Overexpression of each mutant form of desmin individually led to a decrease in muscle contraction generation and morphological changes characteristic of patients with myofibrillar myopathy.	(40)
Huntington’s disease	ААV1/8	Mouse	Brain	ORF of the N-terminal (365 aa) *hHTT* with 18 and 100 CAG repeats under control of CMV enhancer element and the chicken β-actin promoter (Figure 1A).	The overexpression of the N-terminal mutant *hHTT* with 100 CAG repeats led to neuronal death in the striatum and impaired motor functions.	(44)
Anemia of inflammation	ААV8	C57BL/6 mice	Liver	ORF of *mHepc* and *hHEPC* (Figure 1A).	Overexpression of each transgene individually led to microcytosis and hypochromicity. Both models showed weight loss and hair loss.	(46)
Glioblastoma	ААV9	LSL-Cas9 knockin mice	Brain	ORF of Cre-recombinase under control of GFAP promoter and sequences of sgRNAs under control U6 promoter targeting genes associated with cancer (Figure 3).	The joint knockout of genes associated with carcinogenesis led to tumor development in the brains of all experimental animals, followed by their death.	(50)
Lung cancer	AAV9	LSL-Cas9 knockin mice	Lungs	ORF of Cre-recombinase under control of pCBh promoter and sequences of three sgRNA under control of U6 promoter targeting *p53, Kras и Lkb1* (Figure 3).	The simultaneous knockout of the *p53*, *Kras*, and *Lkb1* genes led to tumor development in the lungs of mice, modeling the pathogenesis of the disease in humans.	(55)
Models for virology						
Severe acute respiratory syndrome-related coronavirus 2	ААV6, ААV9	C57BL/6 J and BALB/c mice	Lungs	ORF of *hACE2* under control of CMV enhancer/beta-actin promoter (Figure 1B).	The expression of *hACE2* led to the susceptibility of mice to SARS-CoV-2.	(63)
	AAV-DJ	BALB/c mice	Lungs	ORF of *hACE2* under control of CMV promoter (Figure 1B).	The expression of hACE2 led to the susceptibility of mice to SARS-CoV-2.	(61)
	ААV6.FF	BALB/c и C57BL/6 mice	Lungs	ORF of hACE under control of CASI promoter (Figure 1B).	The expression of hACE2 led to the susceptibility of mice to SARS-CoV-2.	(67)
	AAV2/8	WT and TLR7KO mice	Lungs	ORF of hACE under control of CAG promoter (Figure 1B).	The expression of hACE2 led to the susceptibility of mice to SARS-CoV-2.	(68)
	AAV9	MISTRG6 mice	Lungs	ORF of hАСЕ2 under control of CMV promoter (Figure 1B).	The expression of *hACE2* led to the susceptibility of mice to SARS-CoV-2. The resulting model reproduced persistent disease and increased viral titers compared to other models without human immune cells.	(69)
Hepatitis B	AAV2	C57BL/6 mice	Liver	Replication-deficient HBV genome.	The transgenic cassette carrying the full-length HBV genome facilitated the formation of cccDNA HBV in mouse cells. The resulting mouse model responded to antiviral therapy.	(78)
Severe fever with thrombocytopenia syndrome	AAV2	C57BL/6 mice	Spleen, liver, lungs, kidneys, and small intestine	ORF human DC-SIGN under control of CMV promoter (Figure 1B).	Expression of *hDC-SIGN* in the mouse organism led to susceptibility to the SFTS virus. The generated model demonstrated the pathogenesis of the disease.	(86)

#### 3.1.2 Spinal muscular atrophy type 1

**Figure 2:**
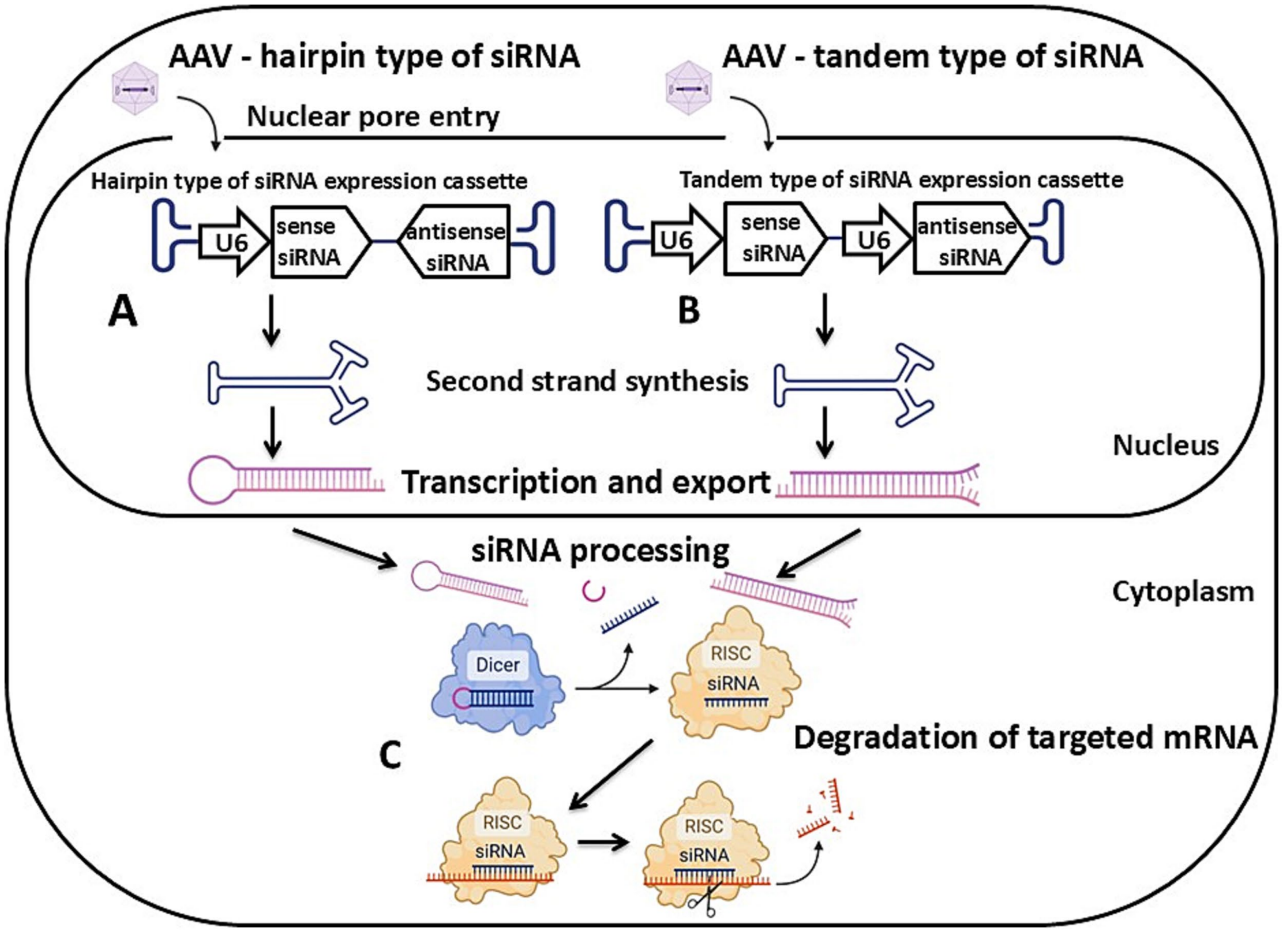
Schematic of AAV vector-mediated transgenesis via the RNA interference mechanism. **(A)** A hairpin type of siRNA expression cassette. **(B)** A tandem type of siRNA expression cassette. **(C)** Degradation of targeted mRNA mediated by the activity of the RNA-induced silencing complex. Detailed description in the text. U6 – U6 promoter, siRNA – small interfering RNA, RISC – RNA-induced silencing complex. Created with BioRender.com.

Type 1 spinal muscular atrophy (SMA) is a neuromuscular disease that leads to early death. Its pathogenesis is characterized by the degeneration of motor neurons in the anterior horns of the spinal cord, resulting in associated muscle atrophy (32). The frequency of type 1 SMA is approximately 1 in 11,000 newborns. The cause of the disease is a reduction or complete absence of *SMN1* gene expression due to mutations. In humans, expression can be partially compensated by the *SMN2* gene, which is absent in other animal species. This leads to difficulties in modeling type 1 SMA in laboratory animals, as the complete absence of the protein results in lethality (33). An animal model of type 1 SMA has been developed in pigs (34) (Table 1 and Figure 2). For this purpose, the shRNA1 sequence targeting porcine *Smn* was cloned into a scAAV9-based plasmid under the control of the H1 promoter, along with GFP, which is driven by the chicken *β*-actin (CBA) promoter. The choice of AAV9 was based on its effectiveness in transducing motoneurons in pigs (35). Preliminary *in vitro* testing of RNA interference effectiveness in pig aorta-derived endothelial cells (PEDSV15) showed a 75% reduction in Smn protein levels. The resulting vectors were administered intrathecally to 5-day-old piglets. 3–4 weeks after the intrathecal injection of scAAV9-shSMN *in vivo*, the piglets developed progressive muscle weakness, particularly in the hind limbs, leading to a complete loss of motor function. The amount of Smn protein in the lumbar spinal cord decreased by 30%. *Smn* mRNA expression was reduced by 73 ± 6% in the motoneurons and by 26 ± 10% in the dorsal horns. Thus, the use of scAAV9-shSMN allowed for the modeling of muscular atrophy-like clinical symptoms characteristic of humans in pigs.

#### 3.1.3 Asthma

Asthma is a chronic non-infectious inflammatory disease of the lower respiratory tract, accompanied by mucus hypersecretion and bronchospasms (36). Some studies have found that the expression of the *CTNNAL1* gene (adhesion molecule catenin alpha-like 1) is reduced in the epithelial cells of the lower respiratory tract in patients suffering from asthma. To confirm the direct link between asthma and the inhibition of the *CTNNAL1* gene, a mouse model with a deficiency of the Ctnnal1 protein in bronchopulmonary tissue was created by transduction with AAV5 carrying small interfering RNA (siRNA) sequences targeting the *Ctnnal1* gene (37) (Table 1 and Figure 2). Ready-made AAV-based vectors were administered intratracheally to eight-week-old female C57BL/6 mice. By the fourth week after infection, the levels of Ctnnal1 protein and mRNA had decreased by 65 and 60%, respectively. Allergen exposure, using house dust mite as the allergen, was carried out on days 35–38 after AAV administration, and led to a significant increase in the levels of interleukin IL-4 and IL-13 in the lungs. As is known, IL-13 and IL-4, released from Th2 cells, are major factors in the development of asthma (38). Overall, the resulting model exhibited symptoms of human asthma: mucus hypersecretion, airway inflammation, and an increase in goblet cell hyperplasia.

#### 3.1.4 Desmin-related myofibrillar myopathy

Desmin-related myofibrillar myopathy is a group of genetic diseases with similar pathological changes in muscle tissue, caused by mutations in the desmin gene. The mutant form of desmin forms abnormal conglomerates, leading to disruptions in the structure of the Z-line in sarcomeres (39). To create a mouse model of this disease, two vectors based on AAV1 were designed, each carrying one variant of the mutant form of desmin: R406W and E413K (40) (Table 1 and Figure 1A). For this purpose, the full-length human desmin cDNAs were cloned into a plasmid between the cytomegalovirus promoter and the human *β*-globin polyA site. To distinguish exogenous desmin from the endogenous form, a 10-amino-acid c-Myc tag was introduced at the 5′-end of the full-length human desmin open reading frame (ORF), the presence of which does not disrupt filament assembly or alter cellular localization (41). All mutant forms of desmin were obtained via site-directed mutagenesis. The resulting vectors, AAV^R406W^ and AAV^E413K^, were injected into the tibialis anterior muscles of 15-week-old female C57BL/6 mice. 4 weeks after injection, the models expressing mutant forms of desmin exhibited morphological changes in muscle fibers (irregular shape and size) characteristic of patients with myofibrillar myopathy. Additionally, a decrease in muscle force generation capacity was detected. Desmin accumulations were identified around the nuclei for R406W and in the subsarcolemmal regions of fibers for E413K. It appears that these accumulations are the cause of Z-line disruptions in sarcomeres. Consequently, both models expressing mutant forms of desmin replicate the pathogenesis of human desmin-related myofibrillar myopathy.

#### 3.1.5 Huntington’s disease

Huntington’s disease (HD) is a hereditary neurodegenerative disorder characterized by neuropsychiatric symptoms, cognitive impairment, and movement disorders (42). Neurodegenerative changes are associated with the expansion of more than 36 CAG repeats in the huntingtin gene (*HTT*). The excessive length of the polyglutamine tract in the huntingtin protein leads to the formation of abnormal neurotoxic conglomerates, which contribute to neuronal degeneration in the striatum and cerebral cortex (43). A model replicating the pathogenesis of Huntington’s Disease was developed to test a drug based on RNA interference (44) (Table 1 and Figure 1A). Two vectors based on mosaic AAV1/8 were developed. One vector carries N-terminal (365 aa) *hHTT* cDNA with 100 CAG repeats, and the other with 18 CAG repeats, both under the control of the CMV enhancer element and the chicken *β*-actin promoter. The resulting AAV vectors were separately injected into the right striata of mice. After 2 weeks, the brains of both groups of mice were analyzed. AAV*Htt*100Q-infected mice showed a reduction in the number of neurons in the dorsal striatum. Nuclear inclusions were detected in striatal and cortical neurons. Additionally, the neurons themselves were smaller in size compared to AAV*Htt*18Q-infected mice, which did not exhibit pathological changes. During behavioral tests (clasping and beam walking), AAV*Htt*100Q-infected mice showed motor function impairments. To suppress the expression of exogenous *HTT*, both groups of mice were given an intrastriatal injection of an siRNA-based drug targeting the *HTT* gene. The exogenous HTT protein level decreased by 66 and 56% in AAV*Htt*100Q- and AAV*Htt*18Q-infected mice, respectively. Moreover, AAVHtt100Q-infected mice exhibited an increase in the number of neurons in the striatum and a reduction in the frequency of nuclear inclusions following this therapy.

#### 3.1.6 Anemia of inflammation

Disruption of the physiological norm of iron levels in the body leads to the development of several diseases, including anemia of inflammation. Hepcidin, a 25-amino acid peptide predominantly expressed in liver cells, plays a key role in iron metabolism. Hepcidin interacts with the iron-export protein ferroportin to control iron release from cellular storage. An excess of hepcidin leads to the blockage of iron export through ferroportin, causing hypoferremia (45). A mouse model simulating anemia of inflammation was created to test the RNA interference-based drug (46) (Table 1 and Figure 1A). For this purpose, two vectors based on AAV8 were constructed. One of them carries the ORF of the mouse hepcidin gene (AAV-mHepc), while the other carries the human gene (AAV-hHEPC). The vectors were administered separately to four-week-old male C57BL/6 mice via the hepatic portal vein. Overexpression of hHepc resulted in dose-dependent hypoferremia and anemia, which developed 2 weeks after transduction. Long-term effect evaluation revealed a decrease in hemoglobin levels 1 month after the AAV vector injection. Further analysis of these models over 4 months showed persistent hypoferremia. Blood analysis revealed the development of microcytosis and hypochromicity. In addition, both models exhibited weight loss and noticeable hair loss, consistent with the known consequences of iron deficiency. To confirm the direct influence of hepcidin on pathogenesis, AAV vectors carrying shRNA were used to selectively suppress *mHepc1* mRNA. Intravenous injection of these constructs into mice led to a reduction in *mHepc1* mRNA in the liver and serum hepcidin levels. Consequently, the serum iron level increased.

#### 3.1.7 Glioblastoma

**Figure 3:**
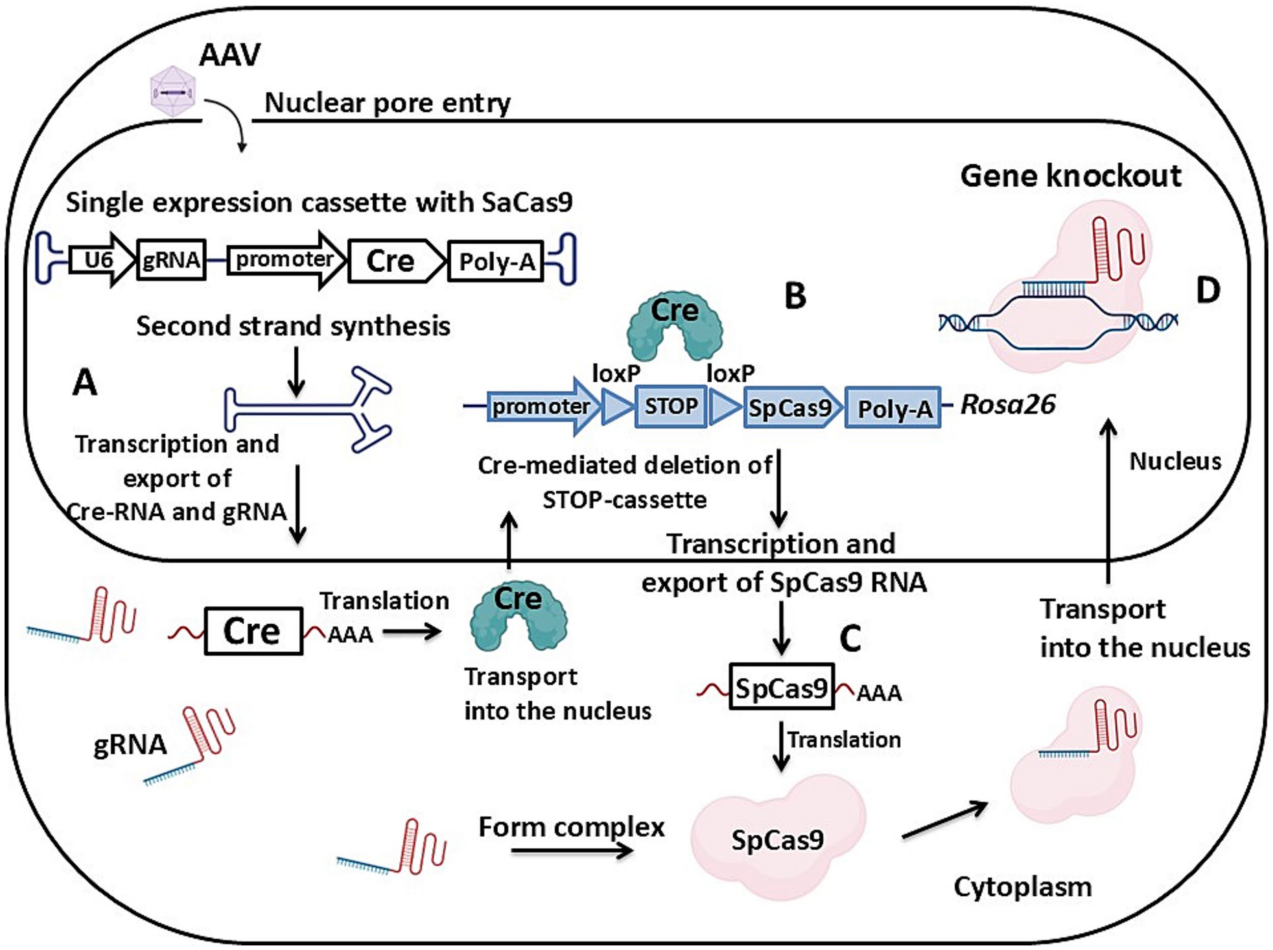
Schematic of AAV vector-mediated transgenesis via the gene knockout mechanism. **(A)** Expression cassette with the Cre recombinase gene and sgRNAs. **(B)** Genome cassette with Cas9 and floxed STOP in *Rosa26* locus. Cre recombinase action is showed. **(C)** Cre-dependent Cas9 expression. **(D)** Knockout of the target gene. Detailed description in the text. U6 – U6 promoter, gRNA – guide RNA, Cre – Cre recombinase, Poly-A – poly-A site, SpCas9 – *Streptococcus pyogenes* Cas9 gene, *Rosa26* – safe harbor in the murine genome highlighted in blue. Created with BioRender.com.

Glioblastoma is a malignant brain tumor that leads to patient death within 12–18 months without proper treatment (47, 48). One of the causes of glioblastoma development in humans is a mutation in the *TRP53* gene (49). To create a mouse model mimicking the development of glioblastoma, several AAV9 vectors were constructed. These vectors carried the Cre recombinase gene controlled by a glial fibrillary acidic protein (GFAP) promoter, along with sgRNA under the control of a U6 promoter, targeting 56 genes commonly mutated in human cancers (50) (Table 1 and Figures 3A–D). The GFAP promoter enabled the induction of Cre recombinase expression in astrocytes only. Notably, each expression cassette contained shRNA for *Trp53* and shRNA for a second gene from the selected panel for co-mutation analysis. *Trp53* was chosen because it is the most frequently mutated gene in carcinogenesis (51, 52). AAV vectors were injected into the lateral ventricle or hippocampus in the brains of LSL-Cas9 mice. This mouse strain carries the Cas9 gene with a loxP-stop-loxP (LSL) cassette at the *Rosa26* locus. Removal of the LSL cassette via Cre recombinase initiates the expression of the Cas protein. Magnetic resonance imaging revealed macrocephaly and tumor development in the brains of mice 4 months after AAV vector injection. Ninety percent of all experimental animals died by day 176 after the AAV vector injection. The remaining animals died by day 299 of the experiment. Histopathology assay showed that the resulting mouse model exhibited features characteristic of human glioblastoma: nuclear aneuploidy and pleiomorphism, dense cellular structure with proliferative spindles, regions of necrosis, hemorrhage, and angiogenesis (53).

#### 3.1.8 Lung cancer

Lung cancer is one of the most common types of cancer leading to death. One form is multi-lesion lung cancer, where multiple tumor lesions form during carcinogenesis (54). To reproduce a similar pathogenesis, a single AAV9 vector was constructed. It carries Cre recombinase gene under the control of the pCBh promoter and three U6-sgRNAs targeting the genes most frequently mutated in lung carcinogenesis: *p53*, *KRAS*, and *LKB1* (55) (Table 1 and Figures 3A–D). This approach allowed for the modeling of simultaneous disruption of multiple gene functions. The AAV vector was injected into 2-3-month-old LSL-Cas9 mice. 2 months after the injection, pathological nodules in the lungs were detected in the mice using micro-computed tomography. Notably, the resulting model exhibited dynamics in tumor volume change and growth rate in different lung segments, reminiscent of the complexity characteristic of human lung tumors.

### 3.2 Infectious diseases models

#### 3.2.1 Severe acute respiratory syndrome-related coronavirus 2

The SARS-CoV-2 virus, first identified in Wuhan, China in 2019, is a member of the *Coronaviridae* family (56). Infection with this virus leads to the development of COVID-19, characterized by damage to the lower respiratory tract (57). A key role in the entry of SARS-CoV-2 into human cells is played by the angiotensin-converting enzyme 2 protein (hACE2). Infection occurs through the interaction between hACE2 and the viral S glycoprotein (58). However, the mouse ortholog mAce2 is not recognized by the SARS-CoV-2 S protein due to amino acid differences between hACE2 and mAce2. Therefore, wild-type mice are not susceptible to SARS-CoV-2 (59, 60). During the ongoing pandemic, there is an urgent need to rapidly develop permissive animal models for detailed study of the new infection and the development of therapeutic drugs and vaccines. Viral vectors are a suitable tool for creating such models under time constraints (61). The initial use of adenovirus as a delivery system for human *hACE2* led to inflammation in the bronchi caused by the vector itself, which distorts the course of the coronavirus infection and the results of experiments when infected with SARS-CoV-2 (62). The application of AAV vectors for mouse transgenesis helped avoid such undesirable effects. Within just 1 month, one of the first mouse models susceptible to SARS-CoV-2 was developed (63) (Table 1 and Figure 1B). For this, 8 to 10-week-old C57BL/6 J mice and BALB/c mice were intravenously injected with AAV6 and AAV9 vectors simultaneously, carrying the ORF of *hACE2* under the control of the CMV enhancer/beta-actin promoter. AAV6 ensured a high level of transduction in murine airway epithelial cells, while AAV9 targeted other extrapulmonary organs. 2 weeks after vector administration, mice from both groups were infected with SARS-CoV-2. The resulting models exhibited weight loss and a high level of SARS-CoV-2 replication in the lungs on days 3–4 post-infection. Analysis of the lungs using hematoxylin and eosin staining showed peribronchial and perivascular inflammatory infiltration with mononuclear cells. This mouse model showed promising results when testing a therapeutic drug based on antibodies against the SARS-CoV-2 spike RBD (RBD-chAb-28 and RBD-chAb-51). Infected mice treated with RBD-chAbs demonstrated a significant reduction in the number of infected cells in the lungs.

Intravenous injection can be replaced with intranasal administration to enhance the transduction efficiency of the lungs. In female BALB/c mice, AAV9, AAV-DJ, and AAV6 carrying the ORF of *hACE2* under the control of the CMV promoter were administered intranasally (61) (Table 1 and Figure 1B). The absence of pathological processes in the lungs was confirmed on the 17th day after the administration of the AAV vector. The expression level of *hACE2* in the lungs was assessed using RT-PCR on the 7th, 14th, and 21st days after transduction. AAV6 demonstrated the lowest transduction efficiency, with *hACE2* mRNA being undetectable in some mice. The expression level of *hACE2* did not significantly differ when delivered by AAV9 and AAV-DJ vectors. Additionally, the expression on the 21st day was the same regardless of the viral titer administered. For the transgenesis of female BALB/c mice, the AAV-DJ serotype was used. It showed higher transduction efficiency compared to AAV6. After infection of the transgenic mice with SARS-CoV-2, a histopathology assay demonstrated inflammatory changes on days 4–7. Hyperplasia of broncho-associated lymphoid tissue and inflammatory infiltration from lymphocytes and histiocytes in the alveoli were observed, indicating the sensitivity of the created model to SARS-CoV-2.

To enhance the ability of AAV6 to transduce tissues in the lower respiratory tract, three mutations were introduced into the canonical AAV6 *cap* gene: F129L, Y445F, and Y731F. The point substitution of tyrosine to phenylalanine prevents ubiquitin-mediated degradation of the vector capsid (64). F129L enhances the efficiency of transducing airway epithelial cells and alveolar type II cells in mice (65). Preliminary testing confirmed a 10-fold increase in the expression of luciferase under the control of the CASI promoter in the lungs, following delivery with the new AAV6.FF (66). This modified capsid was used in the creation of another mouse model susceptible to SARS-CoV-2 (67) (Table 1 and Figure 1B). For this study, 8 to 10-week-old BALB/c and C57BL/6 mice were intranasally administered the AAV6.FF vector carrying the *hACE* ORF, which is controlled by the CASI promoter. Immunofluorescence analysis of *hACE2* expression in the murine lungs showed peak transgene expression on day 10. For this reason, the mice were infected with SARS-CoV-2 on the 10th day after AAV-vector administration. On days 2 and 4 post-infection, a high level of viral replication was observed in the lungs of the mice. On days 8 and 9, some mice exhibited a rapid respiratory rate. Histopathological assays revealed interstitial infiltrates of inflammatory cells, necrosis, type II pneumocyte hyperplasia, vasculitis, and perivasculitis. In addition, an elevated expression level of several genes responsible for immune response formation was detected: granzyme A, granzyme B, *TNF-α*, *IL-6*, *IL-8*, *IL-10*, *TGF-β*, *MCP1*, *VEGF*, *IFN-α*, *IFN-β*, *IFN-γ*. In developing mouse strains susceptible to SARS-CoV-2, pseudotyped AAVs were also used, such as AAV2/8, in which the ITRs were from AAV2 and the capsid from AAV8 (68) (Table 1 and Figure 1B). The AAV2/8 vector, carrying the ORF of *hACE2* under the control of the CAG promoter, demonstrated high transduction efficiency in the lungs of six to eight-week-old WT and TLR7KO male mice following intratracheal and intranasal administration. The cloned *hACE2* contained a FLAG tag at the carboxy terminal for detecting transduction efficiency *in vivo*. On days 6, 10, and 14 after transduction, *hACE2* expression was detected using antibodies to the FLAG tag. Immunoblotting assay showed peak *hACE2* expression on day 14. As a result, mice were infected with SARS-CoV-2 precisely on day 14 after transduction. Alveolar walls were thickened, and mononuclear inflammatory cells were infiltrated around the peribronchiolar and perivascular regions at days 2 and 4 post-SARS-CoV-2 infection. By day 7 post-infection, interstitial pneumonia was infiltrated with immune cells around the perivascular and peribronchiolar regions. These results confirm that the developed mouse model is susceptible to SARS-CoV-2.

Models that replicate human cell-mediated immunity are of particular interest. The use of such mice allowed for a more accurate simulation of COVID-19 symptoms. Based on the humanized strain of MISTRG6 mice, which express human genes *M-CSF*, *GM-CSF*, *IL-3*, *SIRPα*, *ThPO*, and *IL-6*, responsible for the formation of the immune response, a mouse model was developed that mimics the human immune response to SARS-CoV-2 (69) (Table 1 and Figure 1B). For intra-hepatic engraftment, newborn 1-3-day-old pups were injected with 20,000 fetal liver CD34+ human cells. After humanization, eight-week-old mice were intranasally administered an AAV9 vector carrying the ORF of *hACE2* under the control of the CMV promoter. 14 days after the intranasal administration of the AAV vector, the mice were infected with SARS-CoV-2. 2 days post-infection, the MISTRG6-*hACE2* mice demonstrated an accumulation of proteinaceous debris in the alveolar spaces, which is indicative of the exudative phase of infection.

On the 4th day, infiltration of alveolar spaces by fibroblasts, macrophages, and lymphocytes was observed. Between days 14 and 28, the infiltration of the interalveolar space significantly increased. A distinctive feature of this model compared to mice without human immune cells is the elevated titer of SARS-CoV-2. Notably, the presence of human immune cells in MISTRG6-*hACE2* mice caused persistent disease with prolonged weight loss 35 days post-infection. A more severe form of lung pathology was observed, with the pathogenesis occurring in 3 phases characteristic of human COVID-19: exudative, organizing, and fibrotic phases. Thus, the model reproduces key features of COVID-19, including persistent viral RNA, lung pathology with fibrosis, and weight loss (70, 71).

#### 3.2.2 Hepatitis B

The Hepatitis B virus (HBV), a member of the *Hepadnaviridae* family, is a non-cytopathic DNA virus that can cause persistent infection and is associated with a consistent progression to hepatocellular carcinoma and cirrhosis (72). Upon entering the nucleus, the HBV genome undergoes transformation. The relaxed circular DNA is converted into covalently closed circular DNA by host-cell DNA ligases 1 and 3, DNA polymerases *κ* and *α*, and topoisomerases I and II. The cccDNA serves as a template for the synthesis of four different RNAs necessary for the translation of viral proteins and the synthesis of new copies of the HBV genome. One of these RNA molecules, known as pregenomic RNA (pgRNA), is reverse-transcribed back into viral DNA (73). The persistence of cccDNA is crucial for maintaining the full viral life cycle. The use of approved drugs significantly slows down viral replication by inhibiting the viral reverse transcriptase; however, a complete cure for HBV infection is not possible (74). This is because therapeutic drugs are unable to directly affect the cccDNA, which persists in the episome for a long time (75). As a result, prolonged persistence is the cause of HBV reactivation (76). The development of such promising drugs is significantly hindered by the absence of HBV-sensitive animal models that support the formation of cccDNA in cells. This is because the natural hosts of HBV are only humans and NHPs (77). However, the maintenance and use of NHPs are impractical at early stages of research. For this reason, a mouse model harboring cccDNA has been developed (78) (Table 1). Since mice are not susceptible to HBV, an AAV2 vector carrying a replication-deficient HBV genome (AAV-HBV) was used to deliver the viral genome into the cell. After injecting AAV-HBV into the tail vein of six- to eight-week-old C57BL/6 female mice, various forms of HBV DNA were detected in the model animals: relaxed circular DNA, double-stranded linear DNA, AAV-HBV episome, and cccDNA. The presence of cccDNA could be detected up to 44 weeks after injection of AAV-HBV. Chromatin immunoprecipitation assays revealed the binding of cccDNA to histone H3, HBc, and polymerase II, which is characteristic of wild-type cccDNA during HBV infection. Notably, the presence of HBsAg, HBeAg, and HBc proteins was detectable in the model mice up to 66 weeks after the introduction of the AAV vector into the tail vein, confirming the long-term persistence of HBV cccDNA in this model. The mouse model responded to antiviral therapy. 1 week after the injection of AAV-HBV, the mice were administered ETV (Entecavir) or polyinosinic-polycytidylic acid. ETV therapy blocks reverse transcription, limiting the synthesis of rcDNA HBV and consequently the rcDNA conversion necessary for the formation of cccDNA (79). This indicates that in the obtained model, cccDNA was formed as a result of AAV-HBV1.04 episome recombination. Treatment with polyinosinic-polycytidylic acid, which stimulates innate and adaptive immune responses, led to a reduction in HBsAg and HBeAg in the serum of the model mice.

#### 3.2.3 Severe fever with thrombocytopenia syndrome

Severe fever with thrombocytopenia syndrome (SFTS) is an infectious hemorrhagic fever caused by a tick-borne pathogen called the SFTS virus (SFTSV), belonging to the *Phenuiviridae* family. The main clinical symptoms of SFTS in humans and animals are fatal febrile illnesses accompanied by high fever, thrombocytopenia, leukopenia, and multiple organ dysfunction (80). The mortality rate can reach 10–30% of all infection cases (81, 82). For studying SFTSV, IFNAR^−/−^ and STAT2^−/−^ mouse models are used (83, 84). However, these models are not suitable for testing candidate vaccines because they lack immune components. The DC-SIGN receptor (CD-209), located on the surface of human macrophages, monocytes, and dendritic cells, has been identified as one of the receptors necessary for the entry of the SFTS virus (85). To develop a model sensitive to SFTSV, an AAV2 vector carrying the ORF of human *DC-SIGN* under the control of a CMV promoter was used (86) (Table 1 and Figure 1B). 7 days after the injection of the AAV vector into the tail vein of four-week-old C57BL/6 female mice, the expression of *hDC-SIGN* was detected in the spleen, liver, lungs, kidneys, and small intestine. Transduced C57BL/6 mice were intraperitoneally injected with high doses (1 × 10^5^ FAID_50_) of SFTSV. Non-transduced C57BL/6 and IFNAR^−/−^ mice served as controls for SFTSV infection. All IFNAR^−/−^ mice died 4 days post-infection. Infection of the developed mouse model with the SFTS virus led to increased body temperature, weight loss, and thrombocytopenia, with a mortality rate of 12.5% compared to non-transduced C57BL/6 mice and transduced C57BL/6 mice infected with a medium without SFTSV. The study of SFTSV genome distribution in various tissues and organs showed that transduced C57BL/6 mice had higher viral titers compared to non-transduced C57BL/6 mice in the spleen, liver, and lung.

## 4 Discussion

Methods for animal models developing include a wide range of approaches. Approaches based on the use of viral vectors are a promising alternative to classical methods like pronuclear or intracytoplasmic microinjection of the zygote. Transgenesis via viral vectors can be accomplished through various types of medical injections, which is significantly simpler than microinjection into the pronuclei of the zygote, requiring specialized micromanipulation skills. Among viral vectors, rAAV has found widespread application. This is attributed to several advantages over other viral vectors: broad tissue tropism, low immunogenicity, and minimal risk of insertional mutagenesis. This makes the AAV vector a convenient tool for creating animal models. An animal model obtained using the AAV vector should exhibit symptoms of the disease of interest or be sensitive/permissive to a specific pathogen. In existing animal models created with AAV vectors, three molecular biological approaches have been utilized: specific protein production, RNA interference, and gene knockout.

In the animal models obtained so far, different types of promoters have been used: tissue-specific and constitutive. Tissue-specific promoters ensure the expression of the transgene in a specific type of cells or tissue, such as in brain tumor modeling (Figure 3). Constitutive promoters, on the other hand, ensure ubiquitous expression of the transgene regardless of the cell or tissue type. This is particularly useful when a high and stable level of expression is needed in all tissues, for example, in modeling HD or creating virus-sensitive laboratory animals (Figure 1).

The first approach to obtaining animal models through specific protein production allows for the expression of a protein with varying localization within the cell. In the case of modeling proteinopathies or pathological overexpression, such a protein localizes in the cell’s cytoplasm (Figure 1A). For modeling persistent viral infections, such exogenous proteins might be viral proteins. When developing an animal model sensitive to a specific virus, the exogenous receptor protein must be located on the surface of the cell’s cytoplasmic membrane, just like the normal receptor that facilitates viral entry into the cell (Figure 1B). The presence of this new exogenous protein provides sensitivity to human viruses in laboratory animals that are naturally resistant to such pathogens due to the absence of the necessary entrance receptors.

The second approach to obtaining animal models is knockdown or partial suppression of target gene expression using RNA interference. This method provides reversible inactivation or reduction of the target gene’s expression (Figure 2). It is particularly effective when modeling diseases where pathogenesis is characterized by reduced expression rather than a gene knockout. It is worth noting that this approach is also suitable in cases where complete gene inactivation is not feasible due to lethality, such as in modeling SMA type 1 in pigs.

The third approach is an inducible gene knockout in target tissue using genome editing systems. This involves special mouse strains with the gene of Cas9 integrated into the *Rosa26* locus (Figure 3B). In such a strain, the *SpCas9* gene is not typically expressed because a floxed STOP-cassette is situated between the promoter and the ORF. The delivered transgene, in addition to sgRNAs, must also carry the gene for Cre-recombinase (Figure 3A). After AAV transduction, the expressed Cre-recombinase excises the STOP-cassette at the LoxP sites, initiating Cas9 production (Figures 3B,C) and knockout of the target gene with the delivered AAV sgRNAs (Figure 3D). It is important to note that the combination of a specific AAV serotype and promoter allows precise tuning of tissue-specific or widespread gene knockout. The use of a STOP-cassette prevents constant nuclease expression and potential off-target cleavages, which is crucial for the viability of the mouse.

Since each organ is composed of heterogeneous cells with specific functions, there may be a need for transduction of only a portion of them. However, challenges may arise in this case, as the serotype AAV demonstrates tropism to a specific tissue without absolute specificity to the type of its cells. To address such a task, there is a need to modify the capsid proteins of the selected AAV serotype. One approach is to create hybrid serotypes and introduce short peptide sequences into the AAV capsid proteins. Modified serotypes based on AAV9 have been developed this way, demonstrating transduction predominantly into specific types of cells in the nervous system, such as astrocytes (87). Another approach involves attaching a ligand to the capsid protein. This involves disrupting the native motifs of the capsid protein necessary for cell entry and attaching a specific ligand. This ligand ensures cell entry by interacting with a receptor present on the surface of the target cells of a particular organ (88).

Certainly, AAV-based vectors have some drawbacks. One of these is the limited size of the expression cassette, which prevents the delivery of a long ORF with a single vector. However, this can be addressed by using multiple AAV vectors carrying fragments of the same ORF with splicing sites to connect them into a single expression cassette. Another issue may arise when transducing cells with a high proliferation rate. This problem can be resolved by integrating the expression cassette into the genome of such cells. For this, one approach involves using a first AAV vector that carries the expression cassette flanked by transposons, while the second vector carries the transposase gene. Simultaneous transduction with both vectors would result in the integration of the expression cassette into the genome for stable expression of the target gene.

Thus, the use of AAV vectors for transgenesis is a promising direction in developing animal models. Modern molecular-biological and bioinformatics methods enable the production of AAV vectors that offer high transgenesis efficiency in laboratory animals compared to other viral vectors and traditional zygote microinjection methods. There is the ability to regulate the level and tissue-specificity of transgene expression, both during the creation of the expression cassette and in the selection of the AAV serotype, making AAV vectors a versatile tool for transgenesis.
